# Epizootiological aspects of natural nidality of *Ixodes* tick-borne borreliosis in the Moscow region (Russian Federation)

**DOI:** 10.14202/vetworld.2022.213-219

**Published:** 2022-01-30

**Authors:** Almas Mukhametov, Mikhail Osadchuk, Iza Berechikidze, Nikolay Pronkin

**Affiliations:** 1 Department of Technology and Safety of Food Products, Kazakh National Agrarian Research University, Almaty, Kazakhstan; 2 Department of Polyclinic Therapy, I.M. Sechenov First Moscow State Medical University (Sechenov University), Moscow, Russian Federation; 3 Department of Biology and General Genetics, I.M. Sechenov First Moscow State Medical University (Sechenov University), Moscow, Russian Federation; 4 Department of Medical Computer Science and Statistics, I.M. Sechenov First Moscow State Medical University (Sechenov University), Moscow, Russian Federation.

**Keywords:** borreliosis, *Dermacentor reticulatus*, infection, *Ixodes* *ricinus*, *Ixodes* ticks

## Abstract

**Background and Aim:**

At present, tick-borne borreliosis is the most common infectious disease transmitted by ticks in Europe, Asia, and North America. This study aimed to examine the epizootiological aspects of the natural nidality of tick-borne borreliosis in Moscow region (the Russian Federation).

**Materials and Methods:**

A total of 2,537 ticks representing two species were collected, namely, *Ixodes ricinus* and *Dermacentor reticulatus*. The activity, number of ticks, and *Borrelia* infestation rates were investigated during a high season, that is, from early spring to mid-autumn.

**Results:**

In May, amount of *I. ricinus* spp. was found 2.5 times more than those representing *D. reticulatus* spp. (p≤0.01). In June, August, and September, the amount of *I. ricinus* was 9.0 (p≤0.0001), 2.0 (p≤0.05), and 5.0 times higher, respectively, compared to *D. reticulatus*. In the first 10 days of April, the amount of *D. reticulatus* was 3 times higher than that of *I. ricinus* (p≤0.02); in the next 10 days, their amounts were equal (p≥0.05) and in the last 10 days the amount of *I. ricinus* exceeded that of *D. reticulatus* (p≤0.05) by 1.5 times. In general, *Borrelia afzelii*, and *Borrelia garinii*, were detected. In addition, the naturally occurring tick-borne borreliosis pesthole was revealed in the Moscow region.

**Conclusion:**

*Borrelia* infection rates for ticks comprise 30%. An increase in *Borrelia* tick infestation was detected within the vicinity of populated areas. The amount of ticks directly depends on the temperature (20°C-25°C) and moisture (from 50%) values.

## Introduction

An increasing number of borreliosis cases have been recorded to date. This can be explained by the fact that an increasing percentage of ticks are affected by pathogens [1]. Despite the introduction of modern diagnostic methods and the growing number of reports of vector-borne infections, there is still a deficiency of comprehensive understanding of how infections transmitted by ixodid ticks are spread. Only a few infections can be verified, whereas the etiology of other tick-borne infections is unknown or inadequately understood. However, the number of tick bites recorded in health-care facilities is at least 5 times lower than the number of infections transmitted through tick bites [2].

Currently, *Ixodes* tick-borne borreliosis is widespread in the United States, Canada, Russia, and some Western European countries [3,4]. For example, cases are reported from the North West District to the Far East territories in Russia, including Sakhalin [5]. Within the Russia, 7,000-9,000 cases of borreliosis are recorded yearly [6]. Different data on tick borreliosis, ranging between 5% and 90%, are reported in other countries [7]. Recently, new tick-borne pathogens, such as *Rickettsia*, *Ehrlichia*, new forms of *Borrelia*, and *Anaplasma*, have been documented [8]. It indicates that ticks are multifactor objects propagating not one but several diseases simultaneously. This is further aggravated by the fact that various species of ticks spread the pathogens. Among the epidemiological features of borreliosis, residents of urban centers are the largest contributors [9]. This is because citizens are spending a much free time in parks, suburban forests, and summer houses. Other means of tick propagation include various agents with which they enter homes, including flower bouquets, firewood, dogs, fresh hay, and others [10]. Another feature of borreliosis is the age of the primarily affected population – usually, able-bodied people aged between 20 and 59 years [11]. This comprises approximately half of the population. The next risk group consists of up to 30% of children followed by older adults (10-12%) [12]. It is noteworthy that no dependence on professional employment has been established. Particularly, the share of forestry workers, who are one of the primary groups at risk, does not exceed 2-3% among the infected [13].

Approximately 30 species of *Borrelia* are transmitted through mites, but only a few are pathogenic to humans [14]. Its natural hosts are vertebrate wild animals, primarily rodents, and birds. The infection may contribute to the spread of infected ticks during seasonal migration [15]. In Europe and Asia, *Ixodes ricinus* and *Ixodes persulcatus* [16], and in North America, *Ixodes scapularis* and *Ixodes pacificus* [17], are considered agents of infection. However, there are two patterns of infection transmission by the ticks – transphasic and transovarian [18]. The likelihood of being infected through a tick bite is determined by the time the tick has sucked on, which is a significant factor. Furthermore, the infection may come with tick feces if rubbed onto the skin when the bite site is scratched. Besides, borreliosis occurs when people drink unboiled milk of a infected goat. Furthermore, during pregnancy, a *Borrelia* infection may occur and can cause death of the fetus [19].

Subsequent studies have revealed that tick-borne borreliosis pathogens are genetically heterogeneous and can be divided into two main groups. The first group comprises the causal agent of Lyme fever itself, and the second group includes causal agents of recurrent-type fevers [20]. The first group comprises the complex of *Borrelia burgdorferi* sensu lato (s.l.) with 18 genus species [17]. A polymorphic course characterizes Lyme fever (borreliosis). Therefore, no single classification for borreliosis currently exists. Available classifications distinguish between different types and manifestations of the disease. Borreliosis manifests characteristically through clinical signs and the persistence of bacteria. This is usually confirmed by an increase in the titer of certain antibodies. Thus, the following periods of borreliosis duration are distinguished: Acute (disease lasts up to 3 months) and subacute (up to 6 months). Two associated stages are typical of acute borreliosis [21]. The first stage is localized and is characterized by the local spread of infection in the bite site. The second stage is disseminated, implying the propagation of *Borrelia* to the central nervous system, musculoskeletal system. During infection, a process in constant evolution or increasing relapses is possible. The duration of the disease also varies considerably from 6 months to the patient’s lifetime [14].

In general, the typical presence of erythematous rings is found in approximately 70% of all cases and is asymptomatic for the remaining 30% of patients. The most affected organs and systems include the heart, joints, skin, and nervous system. When determining the degree of the disease, the primary focus is on external manifestations, including clinical ones, and organ lesions [22]. Russian statistics indicate that approximately half of all patients (40-51%) have moderate levels of Lyme disease, whereas approximately 1% of all patients have severe forms [15]. An investigation of the potential epizootic aspects of borreliosis is necessary, given the high prevalence of borreliosis in Europe, Asia, and North America [8,11].

For a proper assessment of the epidemiological and epizootic risks of borreliosis, *Borrelia* circulation features in ticks within the study area must be taken into account, including the characteristics of their relations with the reservoir hosts. Therefore, an area combining urbanized landscapes (cities) and forest vegetation zones may serve as a practical model object. The study of the seasonal and territorial dynamics of borreliosis distribution considering the species composition of ticks that transfer it, and the life forms of the ticks themselves (adults and nymphs), allows estimating the probability of Lyme fever infection in a particular region. The resulting data can also be extrapolated to other regions of similar climates. The present study attempt to analyze the peculiarities of the seasonal activity of ticks, the degree of *Borrelia* infection, and the peculiarities of the infection itself; that is, is it a single species of *Borrelia* or is it a hybrid infection? The authors assume that when *Borrelia* tick infestation values are >20%, one can speak of the presence of a tick-transmitted ixodid borreliosis outbreak.

This study aims to investigate the epizootiological aspects of the natural nidality of the ixodic tick-borne borreliosis in Moscow region (the Russian Federation). The study’s objectives included (a) observing seasonal activity of ticks; (b) estimating the extent of tick infestation of *Borrelia* with the identification of the *Borrelia* species; (c) examining *Borrelia* infection in homeless dogs as potential reservoir hosts; and (d) based on the data obtained, making a conclusion whether there is a natural source of tick-borne borreliosis in the Moscow region.

## Materials and Methods

### Ethical approval

The study was approved at the Moscow University Ethics Board meeting (protocol 445-02).

### Study period, location, and sampling

The study was conducted from October 2019 to March 2020 in the Moscow region (Russian Federation). The material was selected from April to October. Two genera of ticks, *Ixodes* and *Dermacentor*, were studied. Besides, the household dogs were examined and more than 2,000 ticks were collected for examinations.

Microclimatic parameters, such as air temperature and humidity levels were considered when collecting materials. Eight routes were chosen for the collection of ticks. In total, approximately 350 km of tick collection roads were plotted during the entire research period.

### Study design

Besides the urban agglomeration of Moscow and satellite cities, the territory under study is quite densely populated (by an average of 40%). Therefore, in this region, visiting suburban forests by urban holidaymakers is a frequent phenomenon. That creates favorable conditions for tick attacks, bites, and possible obtaining *Borrelia* in the blood with subsequent infection with Lyme fever. After all, most ticks are concentrated in forests. The Prioksko-Terrasny Nature Biosphere Reserve (coordinates: 54°54’40.4”N 37°34’18.6”E) was chosen for this study, covering an area of 4.9 thousand hectares. This territory was selected as a model to perform studies on the ecology and biology of ticks, as well as their epizootics. The study results allowed performing an analysis of the infection potential and possible risks within a timber area of the European part of Russia. The data obtained can also be applied to other regions and countries with similar urbanization and forest cover indicators, namely, Ukraine, Belarus, Poland, Germany, Sweden, and Finland. Furthermore, a common database can be created with general parameters (tick infestation with *Borrelia*, indicators of tick activity during the season), which will enable assessing the problem of Lyme fever more broadly than within particular regions.

### Study methods

The ticks were sampled using a method of flag-kilometers, that is, by dragging a piece of flannel cloth over vegetation and recalculating the amount of ticks after every covered kilometer of the study area. The flag was 0.6 m wide and 1 m long. The enumerator moved at a low speed of up to 2 km/h. Ticks that fell on the flag were gathered into tubes. The females were placed in tubes separately from the males to avoid copulation and death shortly afterward. Each tube was marked with road coordinates, vegetation characteristics, and the collector’s name.

A real-time polymerase chain reaction (PCR) was conducted to detect the infestation of ticks with *Borrelia*. This technique allowed 1,139 ticks and their nymphs to be examined. Afterward, the intestinal content was examined, and a portion of the OspA gene, which encodes surface lipoproteins, was magnified. This method was used to detect the deoxyribonucleic acid of *B. burgdorferi* s.l., which is pathogenic to humans. An alternative to this method was applied based on the amplification of the region between the 5S and 23S tandems in the ribosome genes. *Borrelia* species belonging to *B. burgdorferi* s.l. is characterized by duplication of these genes. Because this combination is uncharacteristic of *Enterobacteriaceae* and other *Borrelia* species, it provides a convenient marker for genotyping *Borrelia* species pathogenic to humans. The restriction products were tested through their distribution during electrophoresis, which was conducted in a polyacrylamide gel media. To increase sensitivity, additional tests were conducted using the nested PCR.

Serological tests for evaluating the infestation extent in dogs were conducted. Totally, 115 dogs were taken for examination from the suburban part of Moscow adjoining the forests of Khimki (coordinates: 55°53′21″N, 37°26′42”NW) and Tsavo (coordinates: 55°36’06.8”N, 38°13’02.6”E), including those kept in animal shelters based on a previous agreement with the administration. For dogs’ serodiagnosis of tick borreliosis, immune chemical methods using enzyme-linked immunosorbent assay and WB test systems were used to estimate the total number of boreal cell antigens. Other antigens such as BBK 32, DbpA, VlsE, recombinants, and synthetics were added to increase the system’s sensitivity in case of questionable results. Serum samples from animals previously diagnosed with Lyme disease were used to monitor outcomes. Furthermore, monoclonal antibodies from the reference panel were provided free of charge by Centers for Disease Control and Prevention (USA), and commercial kits derived from the VslE domain were employed.

### Statistical analysis

The results were transferred into Microsoft Excel 2016 (Microsoft, USA) database. In addition, statistical differences between features (tick activity, amount of ticks of different species, ratio of adults and nymphs, and species affiliation to borreliosis pathogen) were analyzed. A correlation analysis was also conducted by evaluating the Pearson correlations between the features. *Borrelia* tick infestation indicators were reported in percentage. The absolute number (total during measurements), the arithmetic mean and, the error of the mean are presented in the graphs and tables. The differences between the signs were determined using Student’s t-test with the significance of differences at p≤0.05.

## Results

**Figure-1:**
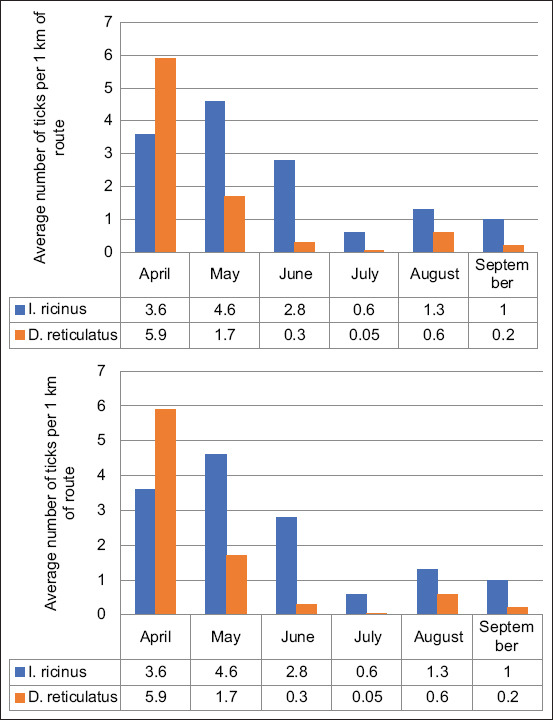
Population dynamics indicators for two species of ticks by months (values per 1 km of road).

Two species of ticks (*I. ricinus* and *D. reticulatus*) that carry borrelia pathogens to humans have been found to reside within the study area. The first species was found shortly after the snow began to melt in the early March, which is consistent with daily mean temperatures above 1°C. In addition, the dynamics of the average annual activity of ticks per month was determined (Figure-1).

For representatives of *D. reticulatus*, the maximum activity and amount were observed in April, whereas the peak of the amount of *I. ricinus*, comes later in May (Figure-1). Both species displayed minimum activity in July. Although the amount of both species began to increase gradually in the following months, it was not as high as in spring, autumn, and late summer (Figure-1). The amounts of both species varied significantly from month to month. In May, amount of *I. ricinus* species was found 2.5 times more as compared to *D. reticulatus* (p≤0.01). In June, this gap increased as *I. ricinus* were found 9.0 times more often (p≤0.0001). In August and September, the amount of *I. ricinus* was 2.0 (p≤0.05) and 5.0 (p≤0.01) times higher, respectively, compared to *D. reticulatus*. It indicates that the activity peak in early spring is typical of *D. reticulatus*. In contrast, predominance of *I. ricinus* was noted in periods of spring to summer (May-June) and summer to autumn (August-September). Therefore, *I. ricinus* can pose a greater threat compared to *D. reticulatus* because ticks of this species are active for a longer period.

**Figure-2:**
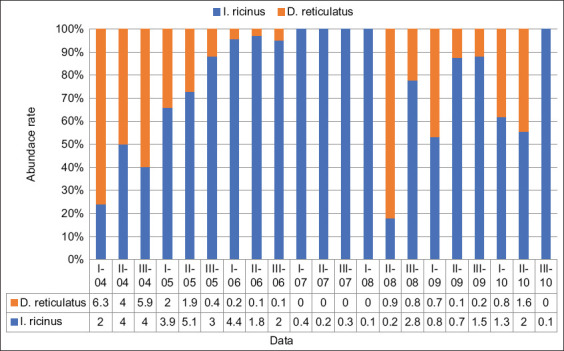
Tick abundance rate by decade for every month between April and October.

A more detailed analysis of the number of ticks of the two species per 10-day period of each month showed that even within 1 month, their numbers may vary significantly (Figure-2).

Specifically, in the first ten days of April, the amount of *D. reticulatus* was 3 times higher than that of *I. ricinus* (p≤0.02), their number was the same during the next ten days (p≥0.05), and in the last ten days of the month, *I. ricinus* increased in number, being 1.5 times more numerous than *D. reticulatus* (p≤0.05). An activity of *I. ricinus* was on a constant increase until the end of the second half of May (Figure-2). In subsequent time intervals, a decrease in the number was observed until the last days of June, with the lowest activity in a second half of August.

The observed increase of *I. ricinus* activity in the second half of June is attributed to precipitation, which encouraged an increase in moisture. This is also associated with the increase in tick activity in the autumn when precipitation occurred after a fairly long interval in September. It has been revealed that *I. ricinus* species can be primarily found near populated areas, whereas this pattern was not found for *D. reticulatus*. On the routes of tick sampling, a smaller number of *D. reticulatus* were found in regions where *I. ricinus* predominated. This can be related not to direct competition but to spatial preferences. Thus, *I. ricinus* prefers forest areas, whereas *D. reticulatus* is prefer more open territories, such as glades, meadows, and forest margins.

Temperature and humidity were positively correlated, especially during spring (0.75, p≤0.05). The peak activity was typical for both tick species at temperatures >20°C and humidity levels of 50% (correlation 0.84, p≤0.01). Simultaneously, there was a decrease in the tick activity with an increase in temperature above 25°C and a decrease in moisture of <50% (correlation −0.67, p≤0.05).

**Figure-3:**
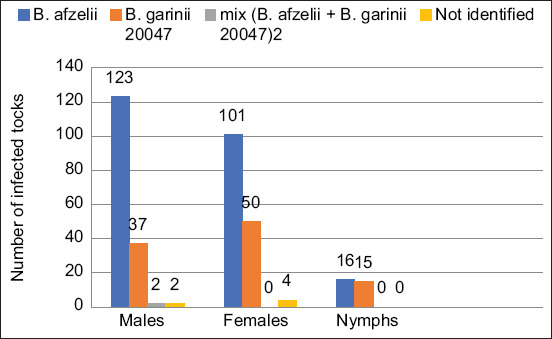
Infection of ticks with *Borrelia*.

*Borrelia* was found in the intestines of a third of the ticks and their nymphs (Table-1). All the detected *Borrelia* species belonged to *B. burgdorferi* s.l. species complex. Two species of *Borrelia*, *Borrelia afzelii* and *Borrelia garinii* 20047, were detected. Therefore, the two *Borrelia* species were found to be present in several ticks simultaneously (Figure-3). It is noteworthy that the ticks most affected by *Borrelia* have been found near populated areas. No reliable differences in *Borrelia* infestation rates were observed between females and males (Table-1).

**Table-1:** Indicators of tick infestation with *Borrelia* in the surveyed area of the Prioksko-Terrasny Biosphere Reserve.

Sex and age of the tick	Number of ticks examined	Number of ticks infected with pathogens+Percentage of the total number
Adult males	511	164+32.1
Adult females	491	155+31.5
Amount	1002	319+31.8
Nymphs	137	31+22.6
Total amount	1139	350+30.7

The number of nymphs infected was 6-8 times lower than the number of adults infected with *Borrelia* in their intestines (p≤0.01). *Ixodes* ticks were also found on dogs. In general, ticks have been reported in 33 dogs. Antibody titers in infected dogs ranged between 1043 and 2529. Interestingly, *Borrelia*-infected dogs were found in the same regions where reports of the levels of *Borrelia* tick infections were highest. Consequently, dogs may play a key role in borreliosis circulation in anthropogenic areas and natural ecosystems.

## Discussion

This study determined that there was a natural source of tick-borne borreliosis. The greatest threat of infection is related to the period of maximum activity of ticks, that is, in early spring and the beginning of autumn. These data are confirmed by several studies [23-25]. With ever-changing weather conditions, infestation periods can vary significantly, in both increasing and decreasing duration. Although tick activity can occur around the clock, they are most aggressive in the morning, from 8 a.m. to 11 a.m. Another activity period occurs in the evening, from 5:00 p.m. to 8:00 p.m. [26]. Ticks have more activity during light rain or cloudy days, and the probability of being attacked by ticks diminishes during heavy rain or hot and sunny weather.

Wild mammals such as rodents, ungulates (deer and roe deer), and birds are known to be *Borrelia* host reservoirs [27]. At the same time, in urbanized landscapes with some forest vegetation, the likelihood that animals having regular contact with humans become major *Borrelia* reservoir hosts are still high. This study shows that homeless dogs can be such hosts for *Borrelia*. Because *Borrelia* is transmitted by *Ixodes* ticks, it is essential to understand the distribution and activity patterns of ticks to evaluate the spread of infection in a proper way. In Europe, two species, *I. persulcatus* and *I. ricinus*, are of major importance and are characterized by maximal aggression toward humans [28]. Simultaneously, these species of ticks are also characterized by a vast range of hosts, that is, they may attack various animals. The so-called transphase transmission of *Borrelia* is known for ticks, indicating that some of these microorganisms penetrate the salivary glands and sexual apparatus of females (according to data, in 5% of ticks) [29]. *Borrelia* transmission from adults to nymphs can range from 40% to 60% in a less frequent transovarian pattern.

Thus, *Borrelia* may circulate in the tick population indefinitely, making the tick population even more dangerous when it comes to the transmission of tick-borne borreliosis. Other data indicate that 7-25% of ticks may be infected by not one but two or three *Borrelia* species [30]. It is also commonly known that a person infected with borreliosis is not a vector of infection and does not pose a danger to others. When infected with multiple species of *Borrelia* and other microorganisms, such as *Ehrlichia*, mixed infections may occur in 17% of cases [31].

It can take from 1 to 2 h between a tick bite and actual infestation of a person with *Borrelia*. Painful sensations occur only 6-8 h after the tick bite, while it usually takes several days for the female tick to saturate with blood [32-34].

The need for the continuation of this research was indicated by the data we obtained on detecting a borreliosis epidemic transmitted by ticks in a densely populated area. Furthermore, it is necessary to create a unified database with geographical reference to the evolution of borreliosis infection rates in the population in different regions per year.

## Conclusion

This study shows that the *Ixodes ricinus-borne* borreliosis prevails on the territory of Prioksko-Terrasny Nature Biosphere Reserve. Among the pathogenic microorganisms, two species of *Borrelia*, *B. afzelii* and *B. garinii*, were detected as pathogens of Lyme fever. *Borrelia* was found exclusively in ticks of *I. ricinus* species, which are the hosts and transmitters of infection. The infection rate of ticks with *Borrelia* is very high and has risen to 30%. An increase in *Borrelia* tick infestation occurred closer to residential areas. A similar pattern was observed for ticks obtained from homeless dogs; the number of animals infected with *Borrelia* (>35%) was highest near populated areas. The number of ticks directly depends on temperature (20°C–25°C) and humidity (more than 50%). The number of ticks decreases significantly, in case of temperature increase or humidity decrease, which generally occurs in the summer.

## Authors’ Contributions

AM: Conceived and designed the study. MO: Collected the data. IB: Data collection and analysis. NP: Performed the analysis. AM, MO, IB and NP: Wrote the manuscript. All authors read and approved the final manuscript.
